# Editorial: Children's Competencies Development in the Home Learning Environment

**DOI:** 10.3389/fpsyg.2021.706360

**Published:** 2021-06-11

**Authors:** Frank Niklas, Caroline Cohrssen, Simone Lehrl, Amy R. Napoli

**Affiliations:** ^1^Department of Psychology, Ludwig-Maximilians-University Munich, Munich, Germany; ^2^Faculty of Education, The University of Hong Kong, Hong Kong, China; ^3^Institute of Psychology, University of Bamberg, Bamberg, Germany; ^4^Department of Child, Youth, and Family Studies, University of Nebraska-Lincoln, Lincoln, NE, United States

**Keywords:** home learning environment, home numeracy environment, home literacy environment, parent-child interaction, children's competencies development

The home learning environment (HLE) is one of the contexts within which young children develop important competencies (e.g., Niklas and Schneider, 2013; Lehrl et al., 2020b), and which affects their long-term development (e.g., Niklas and Schneider, 2017; Lehrl et al., 2020a). Primary caregivers may support children's learning during everyday routine interactions and by shared reading or playing games. Here, it is helpful to differentiate between the home literacy and numeracy environment and their respective associations with children's literacy and numeracy learning (e.g., Niklas and Schneider, 2013, 2014; Lehrl et al., 2014). Further, formal aspects of the HLE include explicit teaching by the primary caregiver, whereas informal aspects of the HLE consist of various activities that foster children's learning, although learning is not the main focus of the activity (cf. Sénéchal and LeFevre, 2002; Skwarchuk et al., 2014). Family intervention programs not only enhance the quality of the HLE, but also support children's competencies development (e.g., Niklas et al., 2016). Meta-analyses show that children who grow up in a high-quality HLE develop greater competencies and are better prepared for school (Sénéchal and Young, 2008; e.g., Mol et al., 2008). In addition, the availability of digital media in many families worldwide offers new possibilities for interventions (e.g., Niklas et al., 2020). The HLE is closely associated with family background variables such as the socioeconomic status (SES) of a family (i.e., families with a high SES tend to provide higher quality HLEs) and the migration background (e.g., Aikens and Barbarin, 2008; Anders et al., 2012; Niklas et al., 2015). Consequently, the HLE acts as a mediator between more distal family characteristics and child outcomes. Further, research indicates that the HLE may not only predict concurrent children's early literacy and numeracy competencies (e.g., Burghardt et al., 2020, Napoli and Purpura, 2018), but also later achievement in school (e.g., Niklas and Schneider, 2017; Lehrl et al., 2020a) as well as more general cognitive abilities (e.g., Howard et al., 2017; Niklas et al., 2018) and socio-emotional outcomes (e.g., Rose et al., 2018; Wirth et al., 2020). Despite the research on the HLE and the findings in recent years, we still do not know how best to operationalize the HLE, through which specific mechanisms the HLE impacts children's learning, and which facets of the HLE are the most important. For instance, in a recent paper by Hornburg et al. (2021), international experts in the field of the home mathematics environment (HME) discussed next steps in the measurement of this construct and concluded that much more work is needed to define and operationalize the HME, so that it can be supported more successfully in research and practise across countries and contexts. Despite more research having been conducted on the home literacy environment than the HME, this issue also applies to the home literacy environment. We are also still in need of successful family intervention approaches that are non-intensive and appeal to all families, independent of their background (e.g., Purpura et al., 2017). Finally, we do not yet know how digital media may be associated with and influence the HLE, or whether there are specific cultural and regional HLE differences.

The present Research Topic entitled “Children's Competencies Development in the Home Learning Environment” thus aims to provide a platform for showcasing the latest research on the HLE and to provide more insight into a construct of important scientific and societal impact. It focuses on empirical research and reviews on children's learning in the context of the HLE. For instance, some of the 23 contributions investigated the different facets of the home literacy and/or numeracy environment (e.g., parental attitudes, parental teaching, frequency, and quality of interactions) and their association with child competencies, whereas others focused on the HLE and children's longer-term development in kindergarten and school, and on intervention effects. Further, some studies compared the HLE across different countries and languages, and some studies focused on digital media usage within the HLE. We ordered the 23 papers according to different criteria and characteristics. Here, we decided to differentiate between articles that focused on both the home literacy and the home numeracy environment (Part 1) and articles that focused on either the home literacy environment (Part 2) or the home numeracy environment (Part 3). Articles in Part 4 compared the HLE across different countries and orthographies and articles in Part 5 analysed digital media within the family context. Within each of these parts, original research articles are presented first and reviews are listed at the end. Finally, within the original research articles in each part, cross-sectional research is presented before manuscripts reporting longitudinal data and manuscripts reporting data on younger children are listed before those reporting data on older children. Table 1 shows an overview of all articles included in this Research Topic and provides information about the article type, the focus variables, the sample (or the studies included in the reviews), the study type (i.e., cross-sectional, longitudinal, or theoretical), and the focus country/ies of the paper. In the following, we briefly highlight key findings from each paper.

**Table 1:** Overview of the articles included in the Research Topic on home learning environment and child outcomes.

**Parts**	**References**	**Article title**	**Article type**	**Focus variables**	**Sample/Included studies**	**Study type**	**Focus countries**
Part 1: research on a general HLE	Napoli et al.	Characteristics related to parent-child literacy and numeracy practises in pre-school	Original research article	Child and family characteristics underlying HLitE and HNE—parent education, home literacy and numeracy practises, parents' beliefs of the importance of literacy and numeracy	199 pre-school children (*M* age = 4.16 years)	C	US
	Susperreguy et al.	Home learning environments of children in Mexico in relation to socioeconomic status	Original research article	SES, HLE (frequency of parental formal home numeracy and literacy activities, parental academic expectations), and children's numeracy performance	173 pre-school children (aged 3–6 years) M age (low SES) = 56.75 months; M age (high SES) = 55.56 months	C	Mexico
	King et al.	Construct-Specific and timing-specific aspects of the home environment for children's school readiness	Original research article	construct- and timing-specific aspects of HLE (stimulation and responsivity construct) for language, maths, and externalising behaviour; school readiness	1,364 children (NICHD data); M age (t1) = 36 months; M age (t2) = 54 months	L	US
Part 2: research on the home literacy environment	Tremblay et al.	From storybooks to novels: a retrospective approach linking print exposure in childhood to adolescence	Brief research report	shared storybook reading in childhood and current print exposure in adolescence, vocabulary, word reading, and spelling skills	45 parent-adolescent dyads; M age (parents) = 47.59, M age (children) = Grades 7–11	C	Canada
	Lau and Richards	Home literacy environment and children's english language and literacy skills in Hong Kong	Original research article	HLitE, children's language and literacy development (English skills)	149 children (M *age* = 59 months)	C	Hong Kong
	Attig and Weinert	What impacts early language skills? Effects of social disparities and different process characteristics of the home learning environment in the first 2 years	Original research article	HLitE, SES, and children's language skills	2,272 families with 2-year-old children (M age= 26 months)	L	Germany
	Linberg et al.	The early years home learning environment–associations with parent-child-course attendance and children's vocabulary at age 3	Original research article	HLitE, attendance of low threshold parent-child-courses, vocabulary development, family background	1,013 children between 2 and 3 years (wave 1: M age = 6.97 months; wave 2: M age = 13.36 months; wave 3: M age = 26.49 months; wave 4: M age = 38.40 months)	L	Germany
	Niklas et al.	The home literacy environment as a mediator between parental attitudes towards shared reading and children's linguistic competencies	Original research article	HLitE and parental attitudes	133 children (average age at t1: 3 years)	L	Germany
	Ebert et al.	Differential effects of the home language and literacy environment on child language and theory of mind and their relation to socioeconomic background	Original research article	home language and literacy environment, language development, ToM, SES	224 pre-school children (3;6 years) M age (t1) = 41.87 months	L	Germany
	Silinskas et al.	Home literacy activities and children's reading skills, independent reading, and interest in literacy activities from kindergarten to grade 2	Original research article	Home Literacy Model and children's engagement in literacy activities at home and at school (children's independent reading, children's interest in literacy, parent teaching)	378 children from pre-school through grade 2 (M age (t1) = 67.7 months)	L	Finland
	Cohen et al.	Longitudinal effects of the family support program Chancenreich on parental involvement and the language skills of pre-school children	Original research article	(1) Attendance of the Chancenreich program and attendance of further educational programs; (2) Family characteristics and attendance rates of program's course; (3) children's vocabulary and grammar development	N(t1)= 182; N(t2)=162 children (T1: *M* age = 41 months, T2: *M* age = 68 months)	L	Germany
	Grolig	Shared storybook reading and oral language development: a bioecological perspective	Review	shared storybook reading and oral language development; interplay of children's, adults' and books' characteristics; HLitE, child care learning environment	Determinants of the shared reading triad's effects on language skills: Bronfenbrenner's bioecological model (1994); shared reading in the HLE (Sénéchal and LeFevre, 2002)	T	–
Part 3: research on the home mathematical environment	Purpura et al.	Examining the factor structure of the home mathematics environment to delineate Its role in predicting pre-school numeracy, mathematical language, and spatial skills	Original research article	Home mathematical environment (direct numeracy, indirect numeracy, spatial) and child outcomes (numeracy, mathematical language, spatial skills)	129 pre-school children (*M*age = 4.71 years)	C	US
	Bachman et al.	Triangulating multi-method assessments of parental support for early math skills	Original research article	Parental support for early math (math talk, home math activities; frequency, type, and content of activities and parental talk)	128 parents (M age = 24–56 year old) of 4-year-old children	C	US
	De Keyser et al.	No association between the home math environment and numerical and patterning skills in a large and diverse sample of 5- to 6-year-olds	Original research article	HNE (home math activities, parental expectations, parental attitudes) and children's mathematical skills	353 pre-school children (M *age* = 70.03 months)	C	Belgium
	Dowker	Home numeracy and pre-school children's mathematical development: Expanding home numeracy models to include parental attitudes and emotions	Review	home numeracy, parental attitudes and beliefs, and children's mathematical performance, gender stereotypes, parental mathematics anxiety on children's anxiety and performance	Home Numeracy Model (Skwarchuk et al., 2014)	T	Germany, Philippines, Ghana, Chile, Italy
	Mutaf-Yildiz et al.	Probing the relationship between home numeracy and chldren's mathematical skills: a systematic review	Systematic review	HNE and children's mathematical skills (formal and informal skills)	37 articles (M age 14–70 months approx.)	T	US, Chile, Germany, China, UK, South Africa, Netherlands, Italy, Belgium, Russia, Canada, Greece
Part 4: research on the HLE across countries and orthograpies	Justice et al.	Parents' growth mindsets and home-learning activities: a cross-cultural comparison of Danish and US parents	Original research article	HLE (family learning activities, learning extensions, parental time investment, parental school involvement) and parental mindset (ability and effort mindset)	497 parents with at least one child aged between 3 and 5 years (*N* danish = 325; *N* USA = 172)	C	Denmark, US
	Inoue et al.	Home literacy environment and early literacy development across languages varying in orthographic consistency	Original research article	HLitE, early literacy development, and varying orthographic constistency (parents'teaching of reading and spelling)	714 first graders into second grade (aged 6+ years) M age different in all countries/samples	L	Canada, Netherlands, Austria, Greece
	Cheung et al.	Home literacy and numeracy environments in Asia	Review	HLE and parents' interest and abilities in Asia/learning- related beliefs and attitudes of parents in Asia, HLitE, and HNE, effectiveness of programs that aim to improve the home learning environment	Studies that have been conducted in different parts of Asia (China, the Philippines, India, Iran, Turkey, and the United Arab Emirates)	T	China, Philippines, India, Iran, Turkey, United Arab Emirates
Part 5: research on the digital HLE	Lehrl et al.	The home learning environment in the digital age–associations between self-reported “analogue” and “digital” home learning environment and children's socio-emotional and academic outcomes	Original research article	Analogue and digital HLE and children's social-emotional and academic competencies	4,914 children aged 0–5 years (Growing up in Germany II data)/M age (toddler sample) = 27.4 months; M age (pre-school) = 58.3 months	C	Germany
	Segers and Kleemans	The impact of the digital home environment on kindergartners' language and early literacy	Original research article	Digital HLitE vs. analogue HLitE, children's language and literacy levels, parental expectations	70 pre-school children (*M* age = 5 years, 11 months)	C	Netherlands
	Dore et al.	Associations between children's media use and language and literacy skills	Original research article	Media use, children's language, and literacy skills	1,583 children from pre-school through 3rd grade	L	US

*HLE, Home Learning Environment; HLitE, Home Literacy Environment; HNE, Home Numeracy Environment; SES, socioeconomic status; ToM, Theory of Mind; c, cross-sectional study; l, longitudinal study; t, theoretical work*.

Part 1 consists of three studies that consider a more general HLE and thus aspects of both the home numeracy and the home literacy environment. In the paper by Napoli et al. the authors examine characteristics of the child and family that relate to the frequency of parent-child literacy and numeracy engagement. Although some characteristics (i.e., parent education and children's age) were related to both literacy and numeracy engagement, parents' beliefs about the importance of literacy were not related to literacy engagement but beliefs about the importance of numeracy were related to numeracy engagement. The second paper by Susperreguy et al. analysed the association of the HLE with children's numeracy outcomes in a sample of 173 Mexican children, aged 3 to 6 years, living in families with high- vs. low-SES. Whereas, parents with high-SES reported a higher frequency of home literacy activities compared with families with low-SES, no such differences were found for numeracy activities. However, home numeracy activities were significantly associated with numeracy skills of children from families with high-SES only. Consequently, the authors' findings indicate that the socioeconomic status of the family should be considered a moderator of the relations between the home numeracy environment and children's early numeracy skills. The third paper by King et al. investigated how time-specific and construct-specific aspects of the home learning environment are related to children's academic skills, and externalising behaviours, using data from the NICHD Study of Early Child Care and Youth Development (*N* = 1,364). They show that although the overall, stable HLE indicator as measured through the Early Childhood Home Observation for Measurement of the Environment Inventory (EC-HOME) at 36 and 54 months (Caldwell and Bradley, 1984) was positively associated with language skills and negatively associated with externalising behaviours, there is also a construct- and time-specific association between the HLE and children's language and mathematics skills. The specific construct “stimulation” was uniquely associated with children's language and mathematics skills, above and beyond the quality of the overall home learning environment.

Part 2 focuses explicitly on the home literacy environment. Here, Tremblay et al. analysed 45 parent-adolescent dyads with a retrospective and current book title and author recognition tests in their brief research report. They found that early reading experiences of the adolescents in this study related to their later reading preferences, which in turn were associated with the current literacy skills of these adolescents. Their findings indicate a long-lasting impact of early shared reading experiences on subsequent interest in reading, as well as on later literacy outcomes well into adolescence. The second paper of Part 2 by Lau and Richards examined the relation between the home literacy environment and ethnic Chinese children's development of English as a second language. Specifically, the authors considered children's English vocabulary, phonological awareness, letter knowledge, and word reading skills. The results indicate a wide range of home literacy support for English language development, and differences in the relations between the home literacy environment and children's skills. The study adds to the growing body of literature examining children's home literacy environment in a multilingual context. In the third paper, Attig and Weinert used data from the German National Educational Panel Study (NEPS) to explore longitudinal relations between the process (i.e., parental interaction behaviour and joint picture book reading) and structural (i.e., socioeconomic status) characteristics of the home environment and children's language skills. They found that families' socioeconomic status was related to each process characteristic, and that several of these characteristics were related to children's vocabulary and grammar skills. In the fourth paper by Linberg et al. data from the German NEPS was also used to investigate the specific relations between quantitative (e.g., frequency of shared book reading) and qualitative (e.g., sensitivity and stimulation during parent-child interaction) indicators of the HLE at age 2 years, as well as the specific impact of attending low threshold parent-child courses in shaping children's vocabulary development between 2 and 3 years of age. The results indicate that the attending parent-child courses enriches both aspects of the HLE, which in turn predict children's vocabulary development. The authors conclude that parent-child courses may be an achieveable target for interventions aimed at very young children. In a longitudinal study by Niklas et al., 133 children aged about 3 years at t1 and parents participated. Here, data related to socioeconomic status, home literacy environment, parental attitudes towards shared reading, and children's linguistic competencies were gathered. The results indicate that parental attitudes towards shared reading seemed to be stable across a 1-year period and that the home literacy environment mediated the effect of parental attitudes towards children's linguistic outcomes. As these attitudes vary in the context of different family socioeconomic backgrounds, the authors conclude that they may also be a good target for interventions. The sixth paper of Part 2 by Ebert et al. examined the specific and differential, longitudinal effects of different facets of the HLE and specific parental mental state language input on language and theory of mind (ToM) development between age 4 and 6 years, using data from 224 monolingual German pre-school children. Here, parental mental state language was defined as high-quality verbal interactions with children on decontextualized topics such as mental states of characters in a book. They found that book exposure and quality of verbal interaction during shared reading is related to both later ToM understanding and language skills. However, the effect of book exposure is mediated by earlier language skills at age 4:6 years. Parental mental state language input was not (additionally) associated with ToM or language skills. However, the effects differed for children from varying SES backgrounds: quality of verbal parent-child interaction and parental mental state language seem to be especially important for children from low SES backgrounds with regard to language and ToM development. Thus, supporting the home language and literacy environment from early on might reduce SES differences not only in language but also in social-cognitive development. In the seventh paper by Silinskas et al., the HOME Literacy Model was tested and expanded within a Finnish sample of children (*N* = 378) transitioning from pre- to primary school over a 3-year period. Through cross-lagged panel analyses they found that both aspects of the HLE, the frequency of shared reading and teaching of reading at home predicted the frequency of children's independent reading 1 year later. Furthermore, they identified children's early literacy skills in pre-school to be a significant predictor for independent reading in Grade 1. Another interesting result is that parents adapted their teaching behaviours to their children's early literacy skills, with showing fewer teaching behaviours for children with advanced skills. However, self-reported interest in reading was not associated with HLE or children's early skills. The results add to the Home Literacy Model through investigating the longitudinal patterns of HLE, early literacy skills, and later independent reading and interest. In the intervention study by Cohen et al., the authors examined the effects of the parent support program Chancenreich on parents' participation in additional educational services and children's vocabulary development and grammar. Parents' participation in the program was related to their later participation in educational services, and to children's vocabulary development between the ages of 3 and 5 years. The study offers initial evidence that family support programs may have longitudinal effects on children's language development. Finally, the review by Grolig investigated the relation between shared storybook reading and oral language development in the home literacy environment and the child care literacy environment. A model is proposed to explain the influence of the interplay between child, adult, and book characteristics on shared reading activities. Drawing on socio-constructivist concepts and Bronfenbrenner's (1994) bioecological model, findings are integrated from psychology, education, and linguistics research and indicate that the effect of shared reading is influenced by characteristics of “literacy agents” as well as the relations between these agents. Further, a combination of questionnaires and recognition tests was found to provide a sufficient evaluation of reading practises in the home literacy environment.

Part 3 of the Research Topic includes manuscripts on the home numeracy (or more general mathematics) environment. The first paper by Purpura et al. examined the factor structure of the HME (general HME factor, direct numeracy, indirect numeracy, and spatial) and tested the association of these factors with children's numeracy, mathematical language, and spatial skills of 129 pre-schoolers from the US. Confirmatory factor analyses indicated that a bifactor model fitted the data best (spatial and a general numeracy factor). In structural equation modelling analyses, only the numeracy factor was able to predict child outcomes when controlling for child and family characteristics. The results highlight the importance of parent-child engagement in specific aspects of mathematics-related activities. In the second article by Bachman et al., the authors used a multimethod approach and assessed mathematics talk during semi-structured observations of parent-child interactions, parent reports on a home math activities questionnaire, and time diaries with a sample of 128 4-year-old children from the US. The findings reveal substantial within-measure variability across all three data sources and some convergence across measures. The authors conclude that this multi-method approach holds great promise for furthering our understanding of when and how parents support early mathematics skills with their pre-school-aged children. The third paper by De Keyser et al. reports that no association was found between the home mathematics environment, and numerical and patterning skills in a diverse sample of 353 children aged 5 to 6 years in Belgium. Neither gender nor family socio-economic moderated the association between the home mathematics environment and children's mathematics skills. Small mathematics-related differences were observed in parental expectations and attitudes. One explanation proposed for these findings is that the pre-school learning environment may play a role due to high participation rates in high quality pre-schools that are fully government subsidised and which include a focus on children's mathematical learning. The fourth paper is a review by Dowker considering the relation between parents' mathematics anxiety and the home numeracy environment of pre-school-aged children. Dowker argues for the importance of a broader definition of the home mathematics environment that includes parent mathematics attitudes in addition to activities. The author also highlights several areas for future research, including broader aspects of mathematics than just numeracy (e.g., measurement) and different aspects of parents' mathematics anxiety. Finally, the review by Mutaf-Yildiz et al. analysed the association of parent-child interactions with numerical content with children's performance in mathematical tasks. Thirty-seven articles were included in their systematic review and the authors found a positive association between home numeracy and children's mathematical skills. Here, more advanced, compared with basic, numeracy interactions were associated with child competencies and most studies used questionnaires, surveyed mothers, analysed a comprehensive total score of mathematical competencies, and focused on formal home numeracy activities. Contradictory results regarding the relation between home numeracy and mathematical skills across studies may be due to differences in these study characteristics.

Part 4 includes articles that compare the HLE across different countries and orthographies. Here, the first paper by Justice et al. analysed parental mindsets (i.e., parental beliefs about the role of ability and effort in learning) and their association with home learning activities across a Danish sample (*N* = 325) and a sample from the US (*N* = 172). The parents of 3- to 5-year-old children in both countries held similar ability and effort mindsets. However, US parents provided more family learning activities, learning extensions, and parental time investment, whereas the Danish parents reported higher levels of school investments. Further, in the US but not in Denmark, higher levels of effort mindset were associated with higher levels of parental time investment. In the second paper by Inoue et al., the authors examined the association between home literacy environment (HLE) and early literacy development in a sample of, on average, 76- to 79-month-old children learning four alphabetic orthographies varying in orthographic consistency (English: *N* = 172; Dutch: *N* = 120; German: *N* = 184; Greek: *N* = 238). The children were tested four times: at the beginning and the end of Grade 1 and Grade 2. In addition, parents reported on parent teaching (PT), shared book reading (SBR), and access to literacy resources (ALR) at the beginning of Grade 1. The findings indicated that SBR did not predict any cognitive or early literacy skills in any language, whereas PT was associated with letter knowledge or phonological awareness in Dutch and Greek only, and ALR was associated with emergent literacy skills in all languages. No specific trend in the role of orthographic consistency in the aforementioned relations emerged. Finally, the review by Cheung et al. synthesises research studies on the home literacy and numeracy environments that have been conducted in different parts of Asia, such as China, the Philippines, India, Iran, Turkey, and the United Arab Emirates. They explore how parents in this part of the world perceive their roles in supporting children's early literacy and numeracy development, through which activities they engage their young children in literacy and numeracy, and how effective intervention programs are that aim to improve the HLEs in Asia. Consistent with the findings from major western studies, the authors report that the home learning environment tends to play a critical role in children's early development also in Asia. However, the authors highlight some findings that seem to be specific to the Asian context: some parents, especially those in East Asia, tend to place greater emphasis on academic achievement and regard it as their own responsibility to support their children's learning at home. In comparison, play is not always favoured as a form of learning, although this view seems to be slightly changing. Furthermore, the role of non-parental household members in fostering children's development is specific to Asian homes, which sometimes might even involve non-family members such as domestic helpers. Furthermore, another challenge might be that in many contexts, children in Asia have to be proficient in languages they do not necessarily speak at home in order to attain educational success—a circumstance which can be observed in many western countries too. Overall the review provides a deep insight in the HLEs in Asian families.

In Part 5, three papers are listed that analyse media usage in the family context and thus a digital HLE. The first paper by Lehrl et al. explores the differentiation of an analogue and a digital HLE and their associations with children's parent-rated academic and socio-emotional outcomes within two age groups in Germany (toddlers and pre-schoolers; total *N* = 4,914). They found that analogue and digital HLE activities are two separate constructs of the HLE which are associated, but not interchangeable. Both dimensions explain individual differences in young children's socio-emotional, practical life, and academic skills; however, these associations are age-specific. For toddlers, only analogue HLE activities were associated with better socio-emotional and practical life skills. For pre-schoolers, digital HLE activities were associated with weaker socio-emotional skills but higher academic skills. However, analogue HLE showed higher effect sizes for the academic outcomes in this age group. The authors conclude that more research is needed on the supporting and detrimental features of the digital HLE. In the second paper by Segers and Kleemans, the authors also tried to differentiate between a digital and an analogue HLE in a sample of 71 families from the Netherlands. Here, the main caregivers of 71 kindergarteners (mean age about 6 years) filled out a questionnaire on the home environment (expectations, activities, and materials), and the children were assessed on language (vocabulary and grammar) and literacy (beginning phoneme awareness, segmentation skill, and grapheme knowledge) skills. Whereas the authors were able to differentiate both forms of the HLE, only the analogue environment was related to children's language abilities (i.e., parental expectations were associated with both language and literacy abilities). Finally, in a longitudinal study by Dore et al., a larger sample from the USA [*N* = 1,583 children (PreK *N* = 238, kindergarten *N* = 466, Grade 1 *N* = 307, Grade 2 *N* = 326, and Grade 3 *N* = 246)] was analysed concerning their media usage and language and literacy skills both at the beginning of the school year and across the school year. The analyses showed that more than 4 h of media usage a day predicted lower literacy gains, but not language gains. However, these effects did not hold in multilevel models. Similarly, no negative associations were found in the single-time models, when controlling for various variables. Further, the findings indicated that younger children are not more vulnerable to detrimental effects. The authors point out that given the concern and popular press coverage around children's media use, it is important to acknowledge non-significant effects in this domain. Their non-significant associations suggest that societal fears around children's media use may be exaggerated. However, characteristics of children's media use, like educational content or adult co-use, may moderate any potential effects.

To sum up, the collection of papers in this Research Topic provides important findings on the complex nature of the HLE and its association with various child outcomes. It assembles 19 empirical articles sampled from 11 nations, namely Austria, Belgium, Canada, Denmark, Finland, Germany, Greece, Hong Kong, Mexico, the Netherlands, and the USA. Further, four reviews are included that either looked at theoretical constructs in the context of the HLE (two reviews) or analysed its association with child outcomes (one review) and across different countries (one review). In these reviews, studies from 18 nations—namely Belgium, Canada, Chile, China, Germany, Ghana, Greece, India, Iran, Italy, the Netherlands, Philippines, Russia, South Africa, Turkey, United Arab Emirates, the UK, and the US—were discussed. Consequently, this Research Topic represents an international and intercultural mix of data sources and perspectives on the HLE.

The articles cover various samples ranging from 45 to almost 5,000 participants (mostly medium-sized samples from *N* = 100 to 500) from early infancy to adolescence (although most focus on pre-school age and kindergarten children). Moreover, this collection grounds a wide range of theoretical approaches, conceptual frameworks, and assessment methods. This leads to highly diverse and nuanced findings concerning the HLE and its association with various child competencies. We hope that the contributions from this Research Topic will spark further scientific work on the HLE and inspire and serve policymakers and practitioners, and that it may lead to new developments concerning the definition, operationalisation, and assessment of the HLE as well as act as a basis for the development of meaningful family interventions.

## Author Contributions

All authors listed have made a substantial, direct and intellectual contribution to the work, and approved it for publication.

## Conflict of Interest

The authors declare that the research was conducted in the absence of any commercial or financial relationships that could be construed as a potential conflict of interest.
